# Gender gap in nonmedical use of anxiolytics among high school adolescents: Tunisia, 2021

**DOI:** 10.1192/j.eurpsy.2023.1121

**Published:** 2023-07-19

**Authors:** S. Rejaibi, A. Silini, M. Zid, R. Mallekh, I. Ben Slema, N. Zoghlami, M. Zribi, S. Ben Youssef, N. Ben Salah, H. Aounallah-Skhiri

**Affiliations:** 1 Department of Epidemiology, National Institute of Health; 2 Medical Faculty of Medicine, Tunis El Manar University; 3 Nutrition Surveillance and Epidemiology department, SURVEN Research Laboratory; 4 Intensive Care Unit department, Center for Urgent Medical Assistance, Tunis, Tunisia

## Abstract

**Introduction:**

Non-Medical Use of Anxiolytics (NMUA) and sedatives is a focus of scientific interest worldwide. In Tunisia, no national epidemiological data related to this issue, are published.

**Objectives:**

We aimed to determine the prevalence of NMUA in Tunisian adolescents and assess specificities from a gender scope.

**Methods:**

Data from the 2021-Mediterranean school Survey on Alcohol and other Drugs (MedSPAD) were used. Based on random sampling method (three-stage stratification), high school teenagers in first and second year of secondary education, were enrolled. Data were collected using a self-administered standardized questionnaire assessing socio-demographic characteristics, and specific questions related to NMUA (among adolescents and close environment), perceived accessibility and initiation age. We studied weighted prevalence estimates of NMUA at least once in a lifetime, presented with 95% Confidence Interval (CI). Epi data software was used for data entry and statistical analysis was performed with STATA software.

**Results:**

The survey included 6.201 adolescents with a mean age of 16.8 years and sex ratio F/M equal to 1.5. Only half of surveyed adolescents, perceived accessibility to NMUA as “impossible” and almost 20% had at least one family member or friend using a nonmedical prescription of anxiolytics. The overall prevalence of NMUA was (8.4%; 95% CI [7.6-9.2]), significantly higher among girls (9.8% Vs 6.1%, p-value<10^-4^). Initiation age was over 13 years for almost 70% of consumers.

**Conclusions:**

Our study highlighted high prevalence of NMUA, mainly among girls. Although COVID mental health impact might have a role in explaining our findings; however, decisions makers should be aware of non-medically prescribed anxiolytics accessibility especially among this vulnerable population. Audit prescription monitoring programs should be reinforced, and multisectoral collaboration should be reinforced to promote adolescents mental well being and avoid falling into addiction trap.

**Disclosure of Interest:**

None Declared

